# Efficacy of urinary [TIMP-2]⋅[IGFBP7], L-FABP, and NGAL levels for predicting community-acquired acute kidney injury in Japanese patients: a single-center, prospective cohort study

**DOI:** 10.1007/s10157-025-02641-8

**Published:** 2025-02-21

**Authors:** Satoshi Inotani, Takeshi Kashio, Yuki Osakabe, Tatsuki Matsumoto, Yoshiki Nagao, Masayuki Ishihara, Hideki Iwata, Keita Mitani, Yutaka Hatakeyama, Taro Horino

**Affiliations:** 1 Department of Endocrinology, Metabolism and Nephrology, Kochi Medical School, Kochi University, Kohasu, Oko-cho, Nankoku, Kochi, 783-8505 Japan; 2 Department of Paediatrics, Kochi Medical School, Kochi University, Kohasu, Oko-cho, Nankoku, Kochi, 783-8505 Japan; 3 Department of Anaesthesiology and Intensive Care Medicine, Kochi Medical School, Kochi University, Kohasu, Oko-cho, Nankoku, Kochi, 783-8505 Japan; 4 Centre of Medical Information Science, Kochi Medical School, Kochi University, Kohasu, Oko-cho, Nankoku, Kochi, 783-8505 Japan

**Keywords:** Acute kidney injury, Biomarker, Insulin-like growth factor-binding protein 7, Liver fatty acid-binding protein, Neutrophil gelatinase-associated lipocalin, Tissue inhibitor of metalloproteinase 2

## Abstract

**Background:**

The combination of urinary tissue inhibitor of metalloproteinase 2 (TIMP-2) and insulin-like growth factor-binding protein 7 (IGFBP7) ([TIMP-2]⋅[IGFBP7]) has emerged as a strong predictor of acute kidney injury (AKI), which is associated with poor outcomes. However, most studies have focused on non-Asian populations, and comparisons of [TIMP-2]⋅[IGFBP7] with other AKI biomarkers in Asian populations have not been performed. Furthermore, no study has examined the efficacy of [TIMP-2]⋅[IGFBP7] for predicting community-acquired AKI.

**Methods:**

We prospectively enrolled adult patients at Kochi Medical School Hospital in Kochi, Japan, and performed a receiver-operating characteristic (ROC) curve analysis to assess the ability of [TIMP-2]⋅[IGFBP7], neutrophil gelatinase-associated lipocalin (NGAL), and liver fatty acid-binding protein (L-FABP) measured at the time of admission to predict AKI.

**Results:**

Of the 170 enrolled patients, 40 (23.5%) developed AKI. Risk factors for AKI development were male sex, history of hypertension, low albumin levels, and high [TIMP-2]⋅[IGFBP7] and NGAL levels. The ROC curve analysis showed that the area under the ROC curve (AUC) of [TIMP-2]•[IGFBP7] for predicting AKI was 0.804 (95% confidence interval [CI], 0.728–0.880); however, the AUCs of L-FABP and NGAL were 0.688 (95% CI, 0.594–0.782) and 0.726 (95% CI, 0.639–0.813), respectively.

**Conclusion:**

Urinary [TIMP-2]⋅[IGFBP7] is a good predictor of community-acquired AKI.

**Supplementary Information:**

The online version contains supplementary material available at 10.1007/s10157-025-02641-8.

## Introduction

Acute kidney injury (AKI) is a syndrome (or, more accurately, a group of syndromes) characterised by an abrupt decrease in glomerular filtration; because of its association with increased morbidity and mortality, AKI is a significant global health concern [1, 2]. AKI can lead to chronic kidney disease (CKD) or end-stage renal disease development, and its incidence is increasing; therefore, its impacts on long-term health and the cost of healthcare are far greater than those previously acknowledged [1, 3]. AKI is an important complication observed in not only patients in the intensive care unit (> 50% of all patients in the intensive care unit) [4] but also patients admitted to general hospital wards (10–15% of all patients who are hospitalised) [5]. Our previous retrospective observational study found that the AKI incidence among Japanese patients who are hospitalised was 7.8% [6]. However, most patients with retrospectively confirmed AKI did not receive treatment at an early stage, presumably because of the difficulty recognising the onset of AKI in patients in general wards and low level of awareness of AKI among clinicians. Because AKI is often diagnosed late or missed entirely, severe consequences occur [7].

Community-acquired AKI (CA-AKI) and hospital-acquired AKI (HA-AKI) have different causes and pathology; therefore, they should be recognized as separate entities. CA-AKI is frequently observed in tropical countries; however, a review of CA-AKI in nontropical countries in Western Europe has not been conducted.

AKI is defined as an increased serum creatinine (SCr) level and decreased urine output over time. The Risk Injury Failure Loss of Kidney Function, End-stage Kidney Disease (RIFLE) criteria first presented diagnostic criteria for AKI in 2004 [8]. These criteria were revised by the Acute Kidney Injury Network (AKIN) in 2007 [9], and they were finally integrated into the Kidney Disease: Improving Global Outcomes (KDIGO) criteria in 2012 [10]. These criteria are used worldwide. Compared to the RIFLE and AKIN criteria, the KDIGO criteria for AKI staging have greater sensitivity for detecting AKI and predicting associated in-hospital mortality [11]. The KDIGO definition of AKI defines stage 1 AKI as an increase of ≥ 0.3 mg/dL in the SCr level within 48 h, an increase of ≥ 1.5 in the SCr level from baseline over the course of 7 days, or urine output < 0.5 mL/kg for 6 h; subsequent stages represent more severe kidney injury [10]. The KDIGO criteria represent the current epidemiological and clinical standards for diagnosing AKI [1, 2]. However, the initiation of therapeutic interventions after confirming changes in SCr levels is often delayed because these changes occur as a result of renal tissue damage.

Early detection of AKI requires careful monitoring of changes in SCr levels and urine output. Nevertheless, in clinical practice, monitoring may not be practical for all patients, especially those hospitalised in a ward other than the intensive care unit. Furthermore, it is nearly impossible to closely monitor outpatients. Changes in SCr levels and urine output are often apparent after renal injury has already occurred [12]. Additionally, the need for at least two measurements to evaluate changes in SCr levels leads to delayed diagnoses. Clinical practice guidelines suggest that risk assessments should be performed to facilitate early AKI detection and prevention [13]. Therefore, a diagnostic method other than monitoring changes in SCr levels and urine volume that allows earlier determination of the risk of AKI is necessary.

Urinary biomarkers that reflect kidney damage, facilitate earlier detection of AKI, enable the recognition of different AKI phenotypes, and allow the evaluation and quantification of the effects of interventions have been validated in various clinical settings [14–17]. The ultimate goal of using biomarkers is the detection of patients at high risk for AKI so that preventive measures can be initiated. A previous study reported that injury and functional biomarkers such as tissue inhibitor of metalloproteinase 2 (TIMP-2), insulin-like growth factor-binding protein 7 (IGFBP7), neutrophil gelatinase-associated lipocalin (NGAL), and L-type fatty acid-binding protein (L-FABP) could be used in combination with clinical information to enhance the ability to diagnose AKI, identify different pathophysiological processes, differentiate the aetiology of AKI, and assess AKI severity [18]. The identification of particular kidney injury biomarkers has enabled a more accurate definition of the pathophysiology, site, mechanism, and severity of injury, thus allowing for a more targeted and individualised treatment plan for patients with AKI [18]. Validated biomarkers can help predict the development or progression of AKI, thus providing opportunities for intervention [18]. Although the identification of AKI biomarkers has progressed, their use in clinical practice is not yet widely accepted. Additionally, the superiority of one biomarker over another as well as the superiority of biomarkers over clinical models remain uncertain. Furthermore, most studies of biomarkers were performed in developed Western countries and focused on hospital-acquired AKI, particularly in intensive care units. To the best of our knowledge, studies of biomarkers in Asian populations are limited, and no study has examined the efficacy of these biomarkers for predicting CA-AKI. Therefore, this study investigated the ability of the combination of urinary TIMP-2 and IGFBP7 ([TIMP-2]⋅[IGFBP7]), NGAL, and L-FABP to predict CA-AKI in a cohort of Japanese adults.

## Methods

### Study design and participants

This prospective observational study was approved by the Institutional Review Board of the Ethics Committee of Kochi Medical School Hospital (IRB approval no.: 28–103) and conducted in accordance with the Declaration of Helsinki (revised in 2013).

Adult patients scheduled for hospitalisation underwent screening before study enrolment at the time of admission to the Department of Endocrinology, Metabolism, and Nephrology of Kochi Medical School Hospital in Kochi, Japan, between 1 January 2018 and 31 July 2022. This study targeted patients who were hospitalised for the first time. All patients included in this study were hospitalised for at least 7 days. Written informed consent was obtained from all enrolled patients. The exclusion criteria were age younger than 18 years, estimated glomerular filtration rate (eGFR) ≤ 30 mL/min/1.73 m^2^, long-term dialysis, prior renal transplantation, and emergency hospital admission.

Urine samples were collected on day 1 of hospitalisation to measure the [TIMP-2]•[IGFBP7], L-FABP, and NGAL levels and characterise the standard reference values of these biomarkers for Japanese patients. Clinical data, including patient demographics, medical history, and SCr levels, were extracted from the hospital records to assess AKI. The eGFRs were calculated based on the SCr data of patients using the Japanese equation for eGFR [19]. CKD was defined according to the KDIGO criteria [20].

### Endpoints

The primary outcome was the occurrence of AKI within 7 days of hospitalisation (i.e., within 7 days of urine collection for biomarker measurements). We defined AKI according to the KDIGO criteria for SCr because urine output data were not available [10]. The baseline SCr value used to diagnose AKI was the SCr value determined based on the results of the blood test performed at the time of admission. These criteria for SCr include an increase of ≥ 0.3 mg/dL in SCr within 48 h or a 1.5-fold increase in SCr within 7 days [10]. We also classified the AKI stage according to the KDIGO criteria for SCr as follows: AKI stage 1, SCr increase of ≥ 0.3 mg/dL or 1.5-times to 1.9-times the baseline value; AKI stage 2, 2.0-times to 2.9-times the baseline value; AKI stage 3, 3.0-times the baseline value, SCr increase to ≥ 4.0 mg/dL, or initiation of renal replacement therapy [10].

### Urine sample analysis

Urine samples were centrifuged after storage at 4 °C for up to 12 h after collection. [TIMP-2]•[IGFBP7] levels in urine supernatants were measured using the VITROS NephroCheck immunoassay and VITROS XT7600 immunodiagnostic system (Ortho Clinical Diagnostics) according to the manufacturer’s instructions. NGAL levels in urine supernatants were measured using the ARCHITECT urine NGAL assay (Abbott), which is a chemiluminescent microparticle immunoassay, according to the manufacturer’s instructions. L-FABP levels in urine supernatants were measured using the LUMIPULSE urine L-FABP assay (Fujirebio), which is a chemiluminescent enzyme immunoassay, according to the manufacturer’s instructions. Certified laboratory technicians blinded to the clinical data performed the analyses. [TIMP-2]⋅[IGFBP7], L-FABP, and NGAL assessments were performed according to the manufacturer’s instructions, and values > 0.3 [ng/mL]^2^/1000, > 8.4 µg/gCr, and > 30.5 µg/gCr, respectively, were considered positive.

### Data collection and covariates

Data were retrospectively collected from the electronic medical records. Trained physicians and research nurses who were unaware of the study and did not participate in patient management or care completed the data input. The following variables were considered covariates: age, sex, body mass index, systolic blood pressure, diastolic blood pressure, history of hypertension, diabetes mellitus, hyperlipidaemia, hyperuricaemia, CKD, chronic heart failure, serum albumin, SCr, eGFR, and urine protein excretion. Clinical and laboratory data, including body mass index, systolic blood pressure, diastolic blood pressure, serum albumin, SCr, eGFR, and urine protein excretion, and urine were collected on day 1 of hospitalisation for biomarker measurements. Patients were considered to have chronic heart failure if it was diagnosed and treated by cardiologists.

### Statistical analysis

All analyses were performed using R software (version 4.1.2; R Foundation for Statistical Computing, Vienna, Austria). Patient demographics are presented as the mean and standard deviation (SD) for continuous variables and as the number of observations with proportions (%) for categorical variables. The Mann–Whitney U test was performed to compare continuous variables between groups, and Fisher’s exact test was used to analyse categorical variables. A logistic regression analysis was performed using AKI onset as the objective variable. Items with fewer than 10 patients were excluded from the regression analysis. Statistical significance was set at* p* < 0.05.

Sensitivity and specificity analyses were performed using receiver-operating characteristic (ROC) curves. The predictive values of these variables for severe AKI were evaluated using ROC curve analyses and the corresponding area under the ROC curve (AUC). DeLong’s test was used to compare AUCs. The diagnostic values of biomarkers were defined as follows: AUC > 0.9, excellent; AUC > 0.75–0.9, good; AUC ≥ 0.50–0.75, poor; and AUC < 0.5, no diagnostic value [21].

## Results

**Fig. 1:**
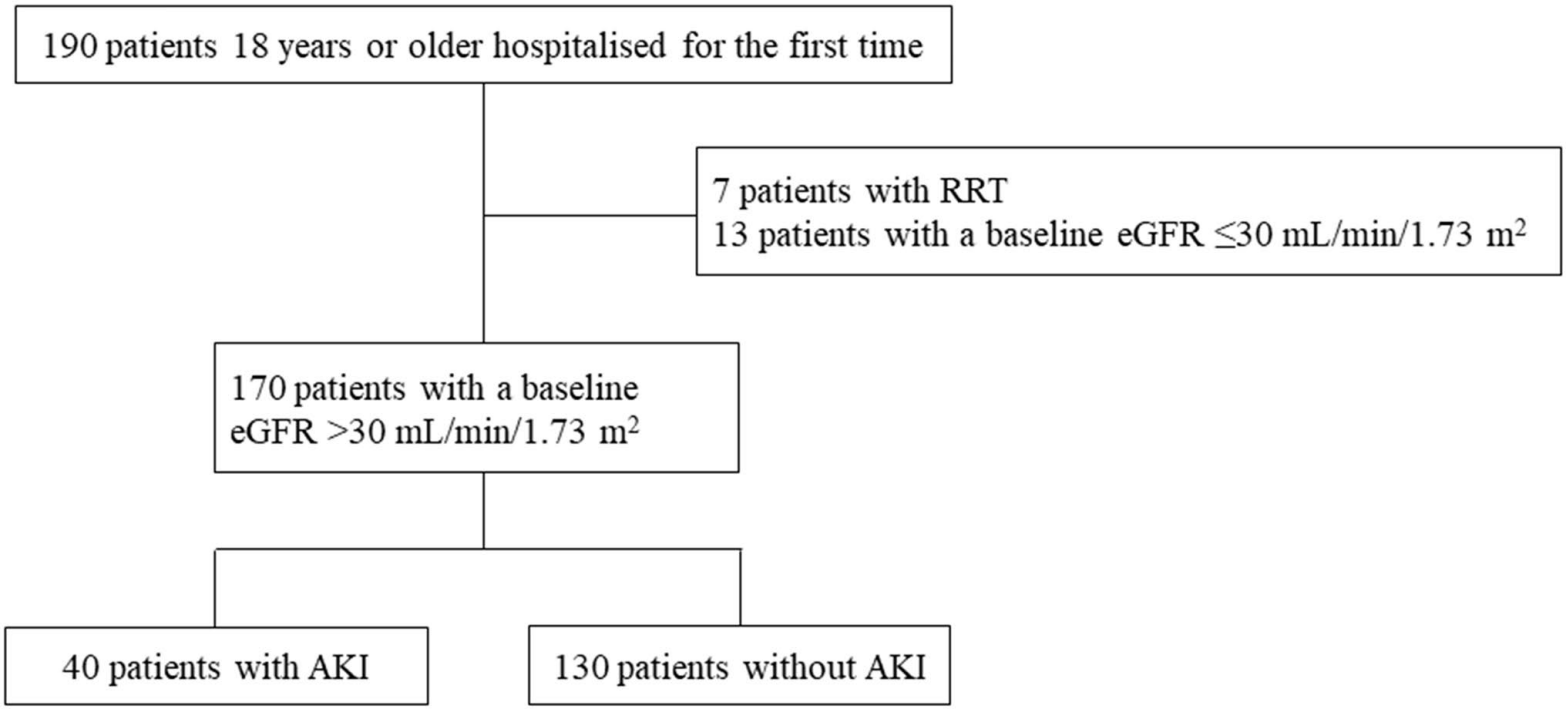
Flowchart illustrating the selection of the study cohort. The inclusion and exclusion criteria and number of participants at each stage are shown. AKI, acute kidney injury; eGFR, estimated glomerular filtration rate; RRT, renal replacement therapy

A flowchart illustrating the cohort selection process used for this study is shown in Fig. 1. We enrolled 190 patients who were admitted to our hospital for the first time. Seven patients were undergoing maintenance dialysis and 13 patients had a baseline eGFR ≤ 30 mL/min/1.73 m^2^. After excluding these 20 patients, 170 patients were included in the final cohort. Table 1 summarises the descriptive statistics of the entire patient group and subgroups with and without AKI, as well as the* p*-values for subgroup differences. The mean age of the entire patient cohort was 61.4 years (± 16.9 years), and 55.2% were women. Of the patients in the final cohort, 40 (23.5%) developed AKI. Thirty-one (18.2%) patients had stage 1 AKI, 7 (4.1%) patients had stage 2 AKI, and 2 (1.1%) patients had stage 3 AKI (Supplementary Fig. 1). Thirty-four (85.0%) patients were diagnosed with AKI 2 days after admission. Six (15.0%) patients were diagnosed with AKI 7 days after admission. Twenty-six (83.8%) patients with stage 1 AKI, six (85.7%) patients with stage 2 AKI, and two (100%) patients with stage 3 AKI were diagnosed more than 2 days after admission. Compared to the non-AKI subgroup, the AKI subgroup included significantly older patients and more men. Liver diseases observed in the patients in this study included viral hepatitis (hepatitis B and C), alcoholic liver disease, nonalcoholic steatohepatitis, and primary biliary cholangitis. Lung diseases observed in patients in this study included interstitial pneumonia, chronic obstructive pulmonary disease, bronchial asthma, tuberculosis, nontuberculous mycobacterial disease, and lung cancer. No differences in body mass index, systolic blood pressure, and diastolic blood pressure were observed between subgroups with and without AKI. Compared to those without AKI, patients with AKI more frequently had hypertension (HT), CKD, and lung disease. Conversely, no differences in the prevalence rates of diabetes mellitus, hyperuricaemia, hyperlipidaemia, chronic heart failure, and liver disease were observed. Patients with AKI had worse baseline values of albumin, SCr, eGFR, and urine protein excretion than those of patients without AKI. Patients with AKI had higher levels of [TIMP-2]⋅[IGFBP7], L-FABP, and NGAL than those of patients without AKI. Risk factors for AKI were male sex, history of HT, low albumin levels, and high [TIMP-2]⋅[IGFBP7] and NGAL levels (Table 2).

**Table 1:** Characteristics of the entire patient group and subgroups with and without AKI

	All patients (n = 170)	With AKI (n = 40; 23.5%)	Without AKI (n = 130; 76.4%)	*p*-value
Age (years), mean (SD)	61.4 (16.9)	66.6 (16.6)	59.8 (16.7)	0.024
Sex, female, n (%)	94 (55.2%)	15 (37.5%)	79 (60.7%)	0.009
BMI (kg/m^2^), mean (SD)	24.1 (5.0)	23.9 (4.5)	24.2 (5.1)	0.798
SBP (mmHg), mean (SD)	127.8 (22.5)	132.1 (26.0)	126.5 (21.2)	0.208
DBP (mmHg), mean (SD)	76.0 (15.5)	74.0 (18.3)	76.6 (14.6)	0.206
HT, n (%)	101 (59.4%)	33 (82.5%)	68 (52.3%)	< 0.001
DM, n (%)	53 (31.1%)	16 (40.0%)	37 (28.4%)	0.168
HL, n (%)	70 (41.1%)	14 (35.0%)	56 (43.0%)	0.364
Hyperuricaemia, n (%)	35 (20.5%)	12 (30.0%)	23 (17.6%)	0.092
CKD, n (%)	112 (65.8%)	35 (87.5%)	77 (59.2%)	< 0.001
CHF, n (%)	23 (13.5%)	9 (22.5%)	14 (10.7%)	0.057
Liver disease, n (%)	39 (22.9%)	10 (25.0%)	29 (22.3%)	0.723
Lung disease, n (%)	41 (24.1%)	15 (37.5%)	26 (20.0%)	0.023
Alb (g/dL), mean (SD)	3.4 (0.8)	2.8 (0.8)	3.6 (0.7)	< 0.001
SCr (mg/dL), mean (SD)	0.9 (0.6)	1.4 (1.1)	0.7 (0.2)	< 0.001
eGFR (mL/min/1.73 m^2^), mean (SD)	70.9 (30.6)	52.1 (28.6)	76.6 (29.0)	< 0.001
UPE (mg/gCr), mean (SD)	213.1 (527.4)	462.7 (940.4)	123.9 (195.0)	< 0.001
UPE ≥ 500 mg/gCr, n (%)	72 (42.3%)	23 (57.5%)	49 (37.6)	0.026
[TIMP-2]⋅[IGFBP7] ([ng/mL]^2^/1000), mean (SD)	0.592 (1.102)	1.327 (1.812)	0.366 (0.617)	< 0.001
[TIMP-2]⋅[IGFBP7] ≥ 0.3 [ng/mL]^2^/1000, n (%)	74 (43.5%)	35 (87.5%)	39 (30%)	< 0.001
L–FABP (µg/gCr), mean (SD)	19.941 (47.871)	35.951 (53.955)	15.014 (44.923)	< 0.001
L–FABP ≥ 8.4 µg/gCr, n (%)	60 (35.2%)	22 (55.0%)	38 (29.2%)	0.003
NGAL (µg/gCr), mean (SD)	131.478 (421.703)	273.220 (576.641)	87.865 (352.565)	< 0.001
NGAL ≥ 30.5 µg/gCr, n (%)	65 (38.2%)	26 (65%)	39 (30%)	< 0.001

*Alb* albumin, *AKI* acute kidney injury, *BMI* body mass index, *CHF* chronic heart failure, *CKD* chronic kidney disease, *DBP* diastolic blood pressure, *DM* diabetes mellitus, *eGFR* estimated glomerular filtration rate, *HL* hyperlipidaemia, *HT* hypertension, *IGFBP7* insulin-like growth factor-binding protein 7, *L-FABP* L-type fatty acid-binding protein, *NGAL* neutrophil gelatinase-associated lipocalin, *SBP* systolic blood pressure, *SCr* serum creatinine, *SD* standard deviation, *TIMP-2* tissue inhibitor of metalloproteinase 2, *UPE* urine protein excretion

**Table 2:** Risk of AKI in our cohort

Covariates	OR	95% CI	*p*-value
Age	0.509	0.149–1.740	0.282
Sex	3.360	1.020–11.100	0.046
BMI	0.389	0.111–1.360	0.139
HT	5.050	1.270–20.100	0.021
DM	0.926	0.273–3.140	0.902
HL	1.130	0.324–3.970	0.844
Hyperuricaemia	1.490	0.391–5.650	0.560
CKD	3.440	0.590–20.100	0.170
CHF	0.614	0.114–3.320	0.571
Liver disease	1.920	0.476–7.780	0.359
Lung disease	2.420	0.685–8.550	0.170
Alb	3.690	1.040–13.000	0.042
eGFR	2.900	0.860–9.770	0.086
UPE	0.833	0.205–3.390	0.798
[TIMP-2]•[IGFBP7]	20.500	5.130–81.700	< 0.001
L-FABP	0.258	0.058–1.130	0.072
NGAL	3.670	1.110–12.200	0.033

The analysis was conducted using age, sex, BMI, Alb, eGFR, UPE, [TIMP-2]•[IGFBP7], L-FABP, NGAL, and comorbid diseases, including HT, DM, HL, hyperuricaemia, CKD, CHF, liver disease, and lung disease, as covariates. The reference values for age, sex, BMI, Alb, eGFR, UPE, [TIMP-2]⋅[IGFBP7], L-FABP, NGAL were younger than 65 years, male, < 25 kg/m^2^, > 3.0 g/dL, > 60 mL/min/1.73 m^2^, < 500 mg/gCr, ≤ 0.3 ng/mL^2^/1000, ≤ 8.4 µg/gCr, and ≤ 30.5 µg/gCr, respectively

*AKI* acute kidney injury, *Alb* albumin, *BMI* body mass index, *CHF* chronic heart failure, *CI* confidence interval, *CKD* chronic kidney disease, *DM* diabetes mellitus, *eGFR* estimated glomerular filtration rate, *HL* hyperlipidemia, *HT* hypertension, *IGFBP7* insulin-like growth factor-binding protein 7, *L-FABP* L-type fatty acid-binding protein, *NGAL* neutrophil gelatinase-associated lipocalin, *OR* odds ratio, *TIMP-2* tissue inhibitor of metalloproteinase 2, *UPE* urine protein excretion

**Fig. 2:**
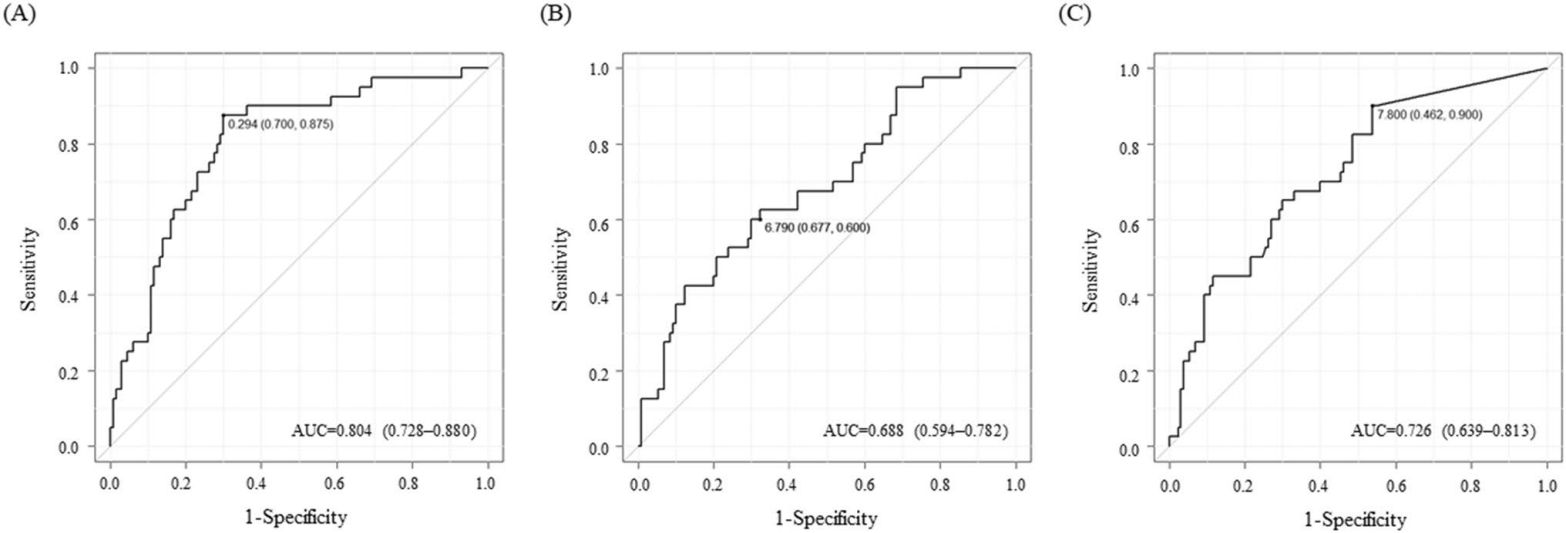
ROC curves for predicting any stage of AKI using [TIMP-2]⋅[IGFBP7] (**A**), L-FABP (**B**), and NGAL (**C**) levels observed in urine samples. AKI, acute kidney injury; AUC, area under the ROC curve; IGFBP7, insulin-like growth factor-binding protein 7; L-FABP, L-type fatty acid-binding protein; NGAL, neutrophil gelatinase-associated lipocalin; ROC, receiver-operating characteristic; TIMP-2, tissue inhibitor of metalloproteinase 2

Figure 2 and Table 3 show the sensitivity and specificity of ROC curves for predicting AKI with [TIMP-2]⋅[IGFBP7], L-FABP, and NGAL, respectively. The AUC values and 95% confidence intervals (CIs) for predicting AKI using [TIMP-2]⋅[IGFBP7], L-FABP, and NGAL were 0.804 (95% CI, 0.728–0.880), 0.688 (95% CI, 0.594–0.782), and 0.726 (95% CI, 0.639–0.813), respectively. When AUCs were compared to determine the usefulness of each biomarker, the *p*-values for [TIMP-2]⋅[IGFBP7] compared with NGAL, [TIMP-2]⋅[IGFBP7] compared with L-FABP, and NGAL compared with L-FABP were 0.168, 0.047, and 0.467, respectively. These results indicated that the diagnostic value of [TIMP-2]⋅[IGFBP7], as assessed by the AUC, was good (0.75–0.9), thus making it valuable for assessing the risk of AKI; however, the diagnostic value of L-FABP and that of NGAL were poor (0.5–0.75). The AUC comparison revealed a significant difference only between [TIMP-2]⋅[IGFBP7] and L-FABP, and that [TIMP-2]⋅[IGFBP7] was superior to L-FABP.

**Table 3:** Prediction of community-acquired AKI using biomarkers and ROC curves

Variable	AUC (95% CI)	Cutoff	Sensitivity	Specificity
[TIMP-2]⋅[IGFBP7] (ng/mL^2^/1000)	0.804 (0.728–0.880)	0.294	0.875	0.700
L–FABP (µg/gCr)	0.688 (0.594–0.782)	6.790	0.600	0.677
NGAL (µg/gCr)	0.726 (0.639–0.813)	7.800	0.462	0.900

*AKI* acute kidney injury, *AUC* area under the ROC curve, *CI* confidence interval, *IGFBP7* insulin-like growth factor-binding protein 7, *L-FABP* L-type fatty acid-binding protein, *NGAL* neutrophil gelatinase-associated lipocalin, *ROC* receiver-operating characteristic, *TIMP-2* tissue inhibitor of metalloproteinase 2

## Discussion

We verified the diagnostic value of [TIMP-2]•[IGFBP7], L-FABP, and NGAL for predicting CSA-AKI using urine samples at the time of hospital admission. Among these biomarkers, [TIMP-2]⋅[IGFBP7] had the best AUC, followed by NGAL and L-FABP; however, only the performance of [TIMP-2]⋅[IGFBP7] was considered good. Therefore, [TIMP-2]⋅[IGFBP7] is useful for predicting CA-AKI.

Our results showed that 23.5% of patients were diagnosed with CA-AKI at the time of hospitalisation. Our cohort included patients who required hospitalisation and admission to the nephrology department; therefore, we speculated that the incidence of CA-AKI among this population was higher than that among the general population. Because patients who required hospitalisation were more likely to have CA-AKI at the time of admission, further verification of our results is required. During outpatient care, measurements of SCr levels were rarely performed more than twice per week. Additionally, patients with CA-AKI do not always present to a hospital for treatment. Therefore, the exact incidence rate of CA-AKI remains unclear. However, our results suggest that the presence or absence of CA-AKI can be determined by measuring biomarkers only once during an outpatient visit, and these biomarker measurements may contribute to future treatment and epidemiological research of CA-AKI.

During this study, the risk of AKI development was assessed using a logistic regression analysis. Our results revealed that male sex, history of hypertension, and low albumin levels were risk factors for CA-AKI. Our results are preliminary because insufficient research of CA-AKI has been performed in nontropical countries, especially Japan; therefore, further epidemiological studies of CA-AKI in the general population are warranted.

Several biomarkers such as TIMP-2, IGFBP7, NGAL, and L-FABP have been investigated to determine their potential roles in the early detection of AKI before SCr levels increase [2]. Kidney damage markers may provide valuable time during which interventions to counter AKI can be initiated, and kidney stress markers may be even more useful than kidney damage markers [2, 22]. Damage markers, such as NGAL and L-FABP, comprise the first generation of AKI biomarkers, which were developed during the past 20 years; however, because of specificity and sensitivity limitations, especially for patients with comorbid conditions, damage markers have been used mainly for research purposes [2]. Stress markers, such as TIMP-2 and IGFBP7, comprise the second generation of AKI biomarkers and were developed during the past 10 years [23]. TIMP-2 is a member of the matrix metalloproteinase family, which can mediate both tissue development and remodeling. IGFBP7 belongs to the IGFBP family and may have a significant biological role in cell proliferation, apoptosis, and senescence. Both TIMP-2 and IGFBP7 have been implicated in G1 cell-cycle arrest [22]. Cell-cycle arrest, which is likely protective for short periods of time, prevents cells from entering the cell cycle during periods of imminent or current injury [22]. However, when cell-cycle arrest is prolonged, cells can transition to a fibrosis phenotype. Studies of AKI demonstrated the role of prolonged cell-cycle arrest in the transition of AKI to CKD [22]. Early activation of these cell-cycle arrest markers functions as an indicator of abnormality. The composite biomarker [TIMP-2]⋅[IGFBP7] demonstrated the ability to predict AKI in large and diverse cohorts of critically ill patients in the Sapphire, Opal, and Topaz studies and better diagnostic accuracy than that of NGAL and L-FABP [23–25]. During the subgroup analyses of the Sapphire and Topaz studies, urinary [TIMP-2]⋅[IGFBP7] predicted AKI with an AUC of 0.84 for patients who underwent cardiac surgery [26]. The aforementioned biomarkers, especially TIMP-2 and IGFBP7, may be able to detect renal stress at a very early stage and shorten the interval between diagnosis and treatment, even when the KDIGO criteria are used. We propose that urinary [TIMP-2]⋅[IGFBP7] may be useful for the diagnosis and treatment of CA-AKI at a very early stage as well.

During the past decade, several studies have reported the efficacy of urinary [TIMP-2]⋅[IGFBP7] for the early detection of AKI. Liu et al. performed a meta-analysis of nine studies that evaluated urinary [TIMP-2]⋅[IGFBP7] using an appropriate study design and concluded that urinary [TIMP-2]⋅[IGFBP7] may be a reliable biomarker that allows early detection of AKI [27]. Of the studies included in the meta-analysis [23–25, 28–33], all included patients in the intensive care unit, five were postsurgical studies, six did not include AKI stage 1, and four included fewer than 100 patients. Large heterogeneity exists between studies, and many have focused on patients in the intensive care unit and included small sample sizes. We previously reported that a high rate of AKI is observed in outpatient clinics, and that the majority outpatients with AKI have stage 1 AKI [6]. Furthermore, we previously reported that each repeated AKI event significantly reduces the eGFR [34]. Therefore, CA-AKI, regardless of its stage, is an important clinical issue that requires early detection. This study focused on CA-AKI at any stage and examined the effectiveness of urinary [TIMP-2]⋅[IGFBP7] for predicting CA-AKI among a large sample; therefore, the results of this study are significant.

Recently, the concept of subclinical AKI was proposed for the early diagnosis of structural changes in renal tissue using biomarkers before declining renal function is indicated by an increased SCr level [18]. The process of diagnosing AKI should be reformulated to include not only renal function markers such as changes in SCr and urine output but also tubular injury markers to improve the characterisation of the AKI phenotype, improve diagnostic accuracy, and detect renal injury before the SCr level increases, thus leading to the identification and treatment of subclinical AKI [18]. Based on our results, cell-cycle arrest marker [TIMP-2]⋅[IGFBP7] could be a powerful tool for diagnosing subclinical CA-AKI.

Our study had some limitations. First, this was a single-centre study; therefore, the generalisability of our findings was limited. Second, hospitalised patients have a wide variety of underlying diseases, and many patients have multiple underlying diseases; therefore, it was difficult to examine the impact of all underlying diseases on biomarkers. A ROC analysis of the main underlying diseases (diabetes mellitus, HT, and CKD) was performed; however, their AUCs were not significant (Supplementary Figs. 2–4). Third, during this study, many patients were hospitalised or presented for treatment because of reasons other than renal disease. Many patients also presented to other hospitals as outpatients after discharge. Therefore, the long-term renal prognosis and life prognosis could not be adequately monitored. Similar to our previous study [34], the majority of patients with CA-AKI had stage 1 AKI, which may have been the reason why no deaths occurred and no patients required short-term renal replacement therapy. However, with AKI, even minor changes in creatinine can affect the long-term prognosis. Further research of the long-term prognosis of CA-AKI determined using AKI biomarkers, especially [TIMP-2]⋅[IGFBP7], is necessary. Finally, this study aimed to verify the validity of using biomarkers to predict the onset of CA-AKI, but it did not focus on CA-AKI alone. CA-AKI is a syndrome that comprises AKI and has a wide range of causes; therefore, further investigations of CA-AKI with different causes are required. Additional studies of the pathology and epidemiology of CA-AKI are also necessary. The results of this study may lead to further research of CA-AKI detection using AKI biomarkers, especially [TIMP-2]⋅[IGFBP7].

## Conclusions

Among the urinary biomarkers evaluated, [TIMP-2]⋅[IGFBP7] exhibited the best predictive ability for CA-AKI. Our findings indicate that [TIMP-2]⋅[IGFBP7] is particularly useful for diagnosing CA-AKI in Japanese populations. Further research of CA-AKI and its identification in patients using [TIMP-2]⋅[IGFBP7] should be performed.

## Supplementary Information

Below is the link to the electronic supplementary material.

Supplementary file1 Supplementary Fig. 1 Incidence, stage, and timing of the AKI diagnosis during this study. (A) Incidence of each stage of AKI. (B) Timing of the AKI diagnosis. (C) Timing of the AKI diagnosis for each stage of AKI. AKI, acute kidney injury (TIF 1642 KB)

Supplementary file2 Supplementary Fig. 2 ROC curves for predicting DM using [TIMP-2]⋅[IGFBP7] (A), L-FABP (B), and NGAL (C) levels observed in urine samples. AUC, area under the ROC curve; DM, diabetes mellitus; IGFBP7, insulin-like growth factor-binding protein 7; L-FABP, L-type fatty acid-binding protein; NGAL, neutrophil gelatinase-associated lipocalin; ROC, receiver-operating characteristic; TIMP-2, tissue inhibitor of metalloproteinase 2 (TIF 3560 KB)

Supplementary file3 Supplementary Fig. 3 ROC curves for predicting HT using [TIMP-2]⋅[IGFBP7] (A), L-FABP (B), and NGAL (C) levels observed in urine samples. AUC, area under the ROC curve; HT, hypertension; IGFBP7, insulin-like growth factor-binding protein 7; L-FABP, L-type fatty acid-binding protein; NGAL, neutrophil gelatinase-associated lipocalin; ROC, receiver-operating characteristic; TIMP-2, tissue inhibitor of metalloproteinase 2 (TIF 3533 KB)

Supplementary file4 Supplementary Figure 4 ROC curves for predicting CKD using [TIMP-2]⋅[IGFBP7] (A), L-FABP (B), and NGAL (C) levels observed in urine samples. AUC, area under the ROC curve; CKD, chronic kidney disease; IGFBP7, insulin-like growth factor-binding protein 7; L-FABP, L-type fatty acid-binding protein; NGAL, neutrophil gelatinase-associated lipocalin; ROC, receiver-operating characteristic; TIMP-2, tissue inhibitor of metalloproteinase 2 (TIF 4010 KB)

## Data Availability

The ethics committee of Kochi Medical School Hospital restricts the sharing of clinical data because these are confidential and subject to general data protection regulations. The datasets used and/or analysed during the current study are available from the corresponding author upon reasonable request.
