# Supplementary figures and images for: Efficacy of urinary [TIMP-2]⋅[IGFBP7], L-FABP, and NGAL levels for predicting community-acquired acute kidney injury in Japanese patients: a single-center, prospective cohort study
Source: Clin Exp Nephrol. 2025 Feb 21;29(7):928–36. doi: 10.1007/s10157-025-02641-8 (PMC12204939; doi:10.1007/s10157-025-02641-8)

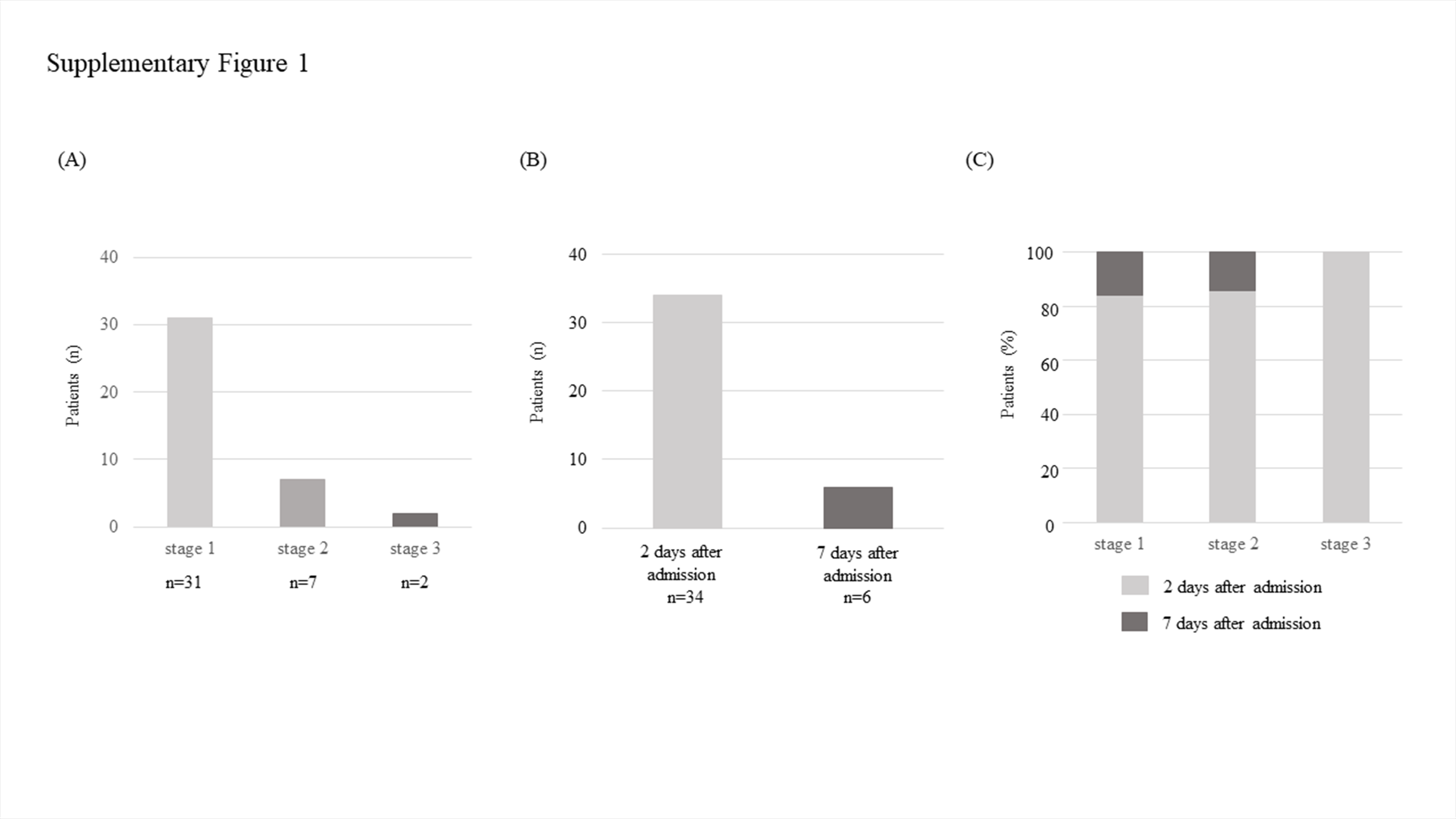

Supplement: Supplementary file 1 — Supplementary file1 Supplementary Fig. 1 Incidence, stage, and timing of the AKI diagnosis during this study. (A) Incidence of each stage of AKI. (B) Timing of the AKI diagnosis. (C) Timing of the AKI diagnosis for each stage of AKI. AKI, acute kidney injury (TIF 1642 KB) [file 10157_2025_2641_MOESM1_ESM.tif]

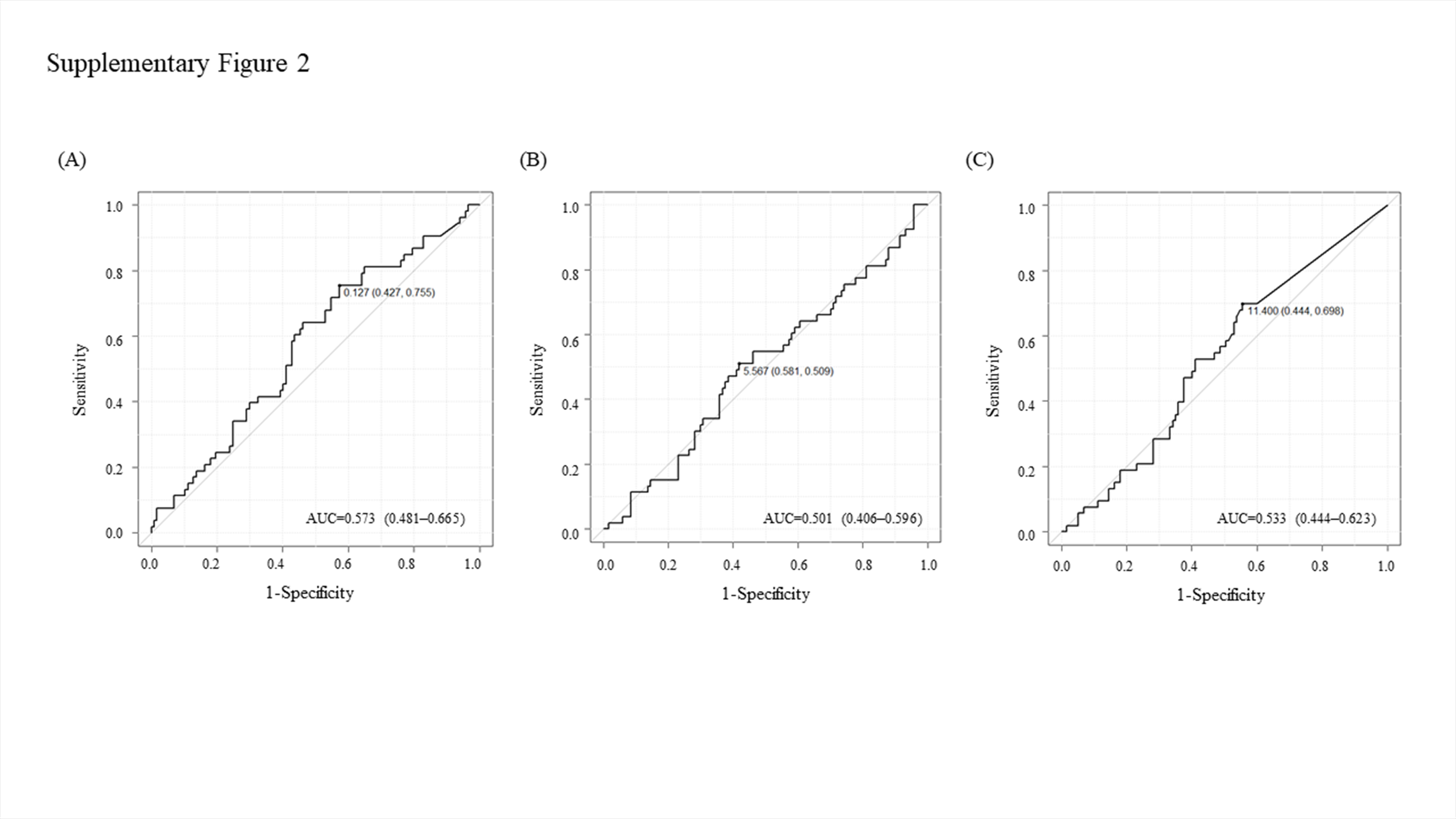

Supplement: Supplementary file 2 — Supplementary file2 Supplementary Fig. 2 ROC curves for predicting DM using [TIMP-2]⋅[IGFBP7] (A), L-FABP (B), and NGAL (C) levels observed in urine samples. AUC, area under the ROC curve; DM, diabetes mellitus; IGFBP7, insulin-like growth factor-binding protein 7; L-FABP, L-type fatty acid-binding protein; NGAL, neutrophil gelatinase-associated lipocalin; ROC, receiver-operating characteristic; TIMP-2, tissue inhibitor of metalloproteinase 2 (TIF 3560 KB) [file 10157_2025_2641_MOESM2_ESM.tif]

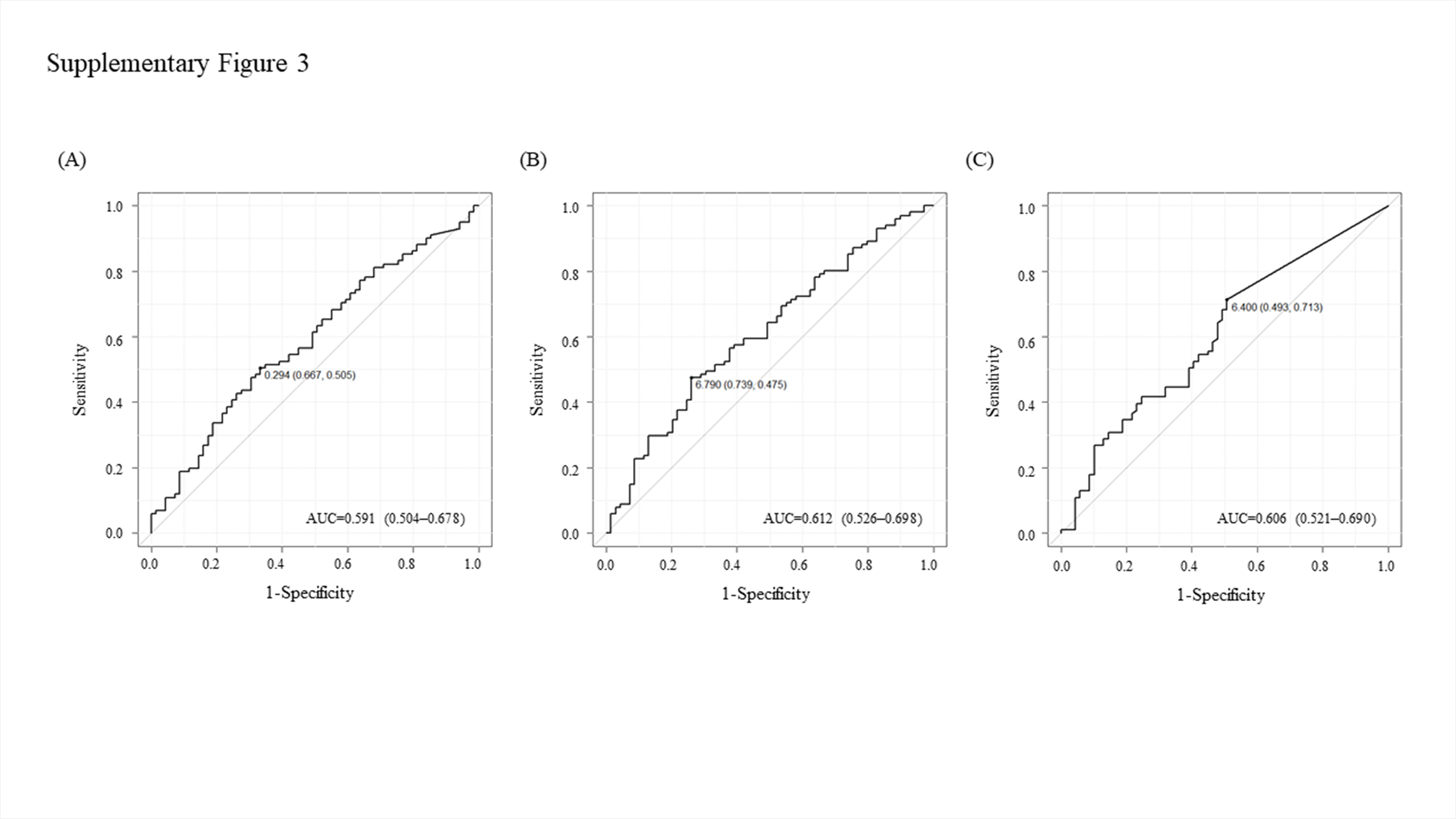

Supplement: Supplementary file 3 — Supplementary file3 Supplementary Fig. 3 ROC curves for predicting HT using [TIMP-2]⋅[IGFBP7] (A), L-FABP (B), and NGAL (C) levels observed in urine samples. AUC, area under the ROC curve; HT, hypertension; IGFBP7, insulin-like growth factor-binding protein 7; L-FABP, L-type fatty acid-binding protein; NGAL, neutrophil gelatinase-associated lipocalin; ROC, receiver-operating characteristic; TIMP-2, tissue inhibitor of metalloproteinase 2 (TIF 3533 KB) [file 10157_2025_2641_MOESM3_ESM.tif]

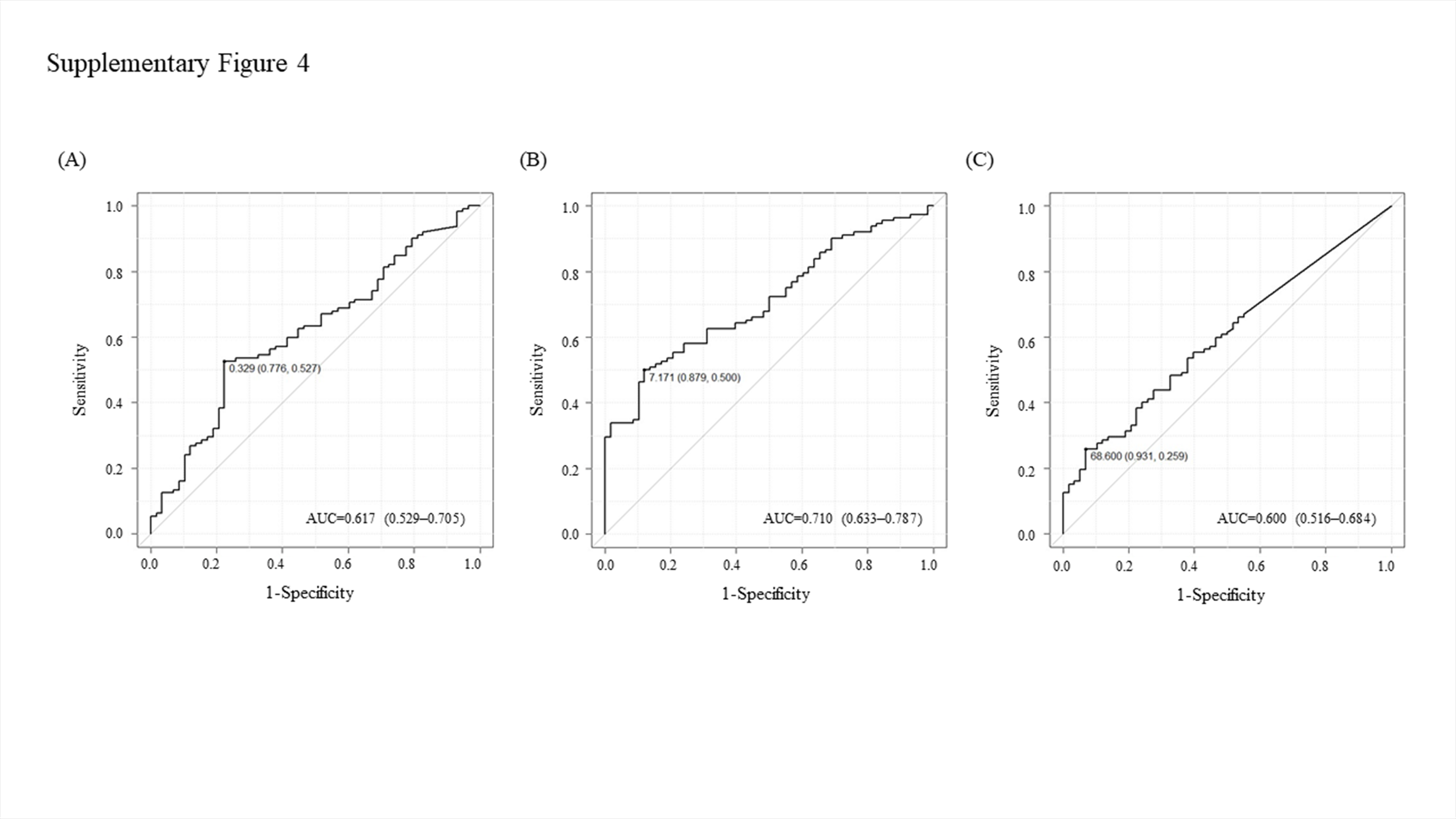

Supplement: Supplementary file 4 — Supplementary file4 Supplementary Figure 4 ROC curves for predicting CKD using [TIMP-2]⋅[IGFBP7] (A), L-FABP (B), and NGAL (C) levels observed in urine samples. AUC, area under the ROC curve; CKD, chronic kidney disease; IGFBP7, insulin-like growth factor-binding protein 7; L-FABP, L-type fatty acid-binding protein; NGAL, neutrophil gelatinase-associated lipocalin; ROC, receiver-operating characteristic; TIMP-2, tissue inhibitor of metalloproteinase 2 (TIF 4010 KB) [file 10157_2025_2641_MOESM4_ESM.tif]
